# A Nonlinear Error Compensation Method for Heterodyne Interferometry Based on Self-Supervised Physics-Informed Neural Networks with Frequency-Domain Priors

**DOI:** 10.3390/s26103000

**Published:** 2026-05-10

**Authors:** Yao Wang, Hongyu Sun, Jiakun Li, Chenlong Ma, Ying Zhang, Xiao Wang, Qibo Feng

**Affiliations:** Key Lab of Luminescence and Optical Information, School of Physical Science and Engineering, Beijing Jiaotong University, Beijing 100044, China; yaowang1232021@163.com (Y.W.); shymanuscript@163.com (H.S.); jkli@bjtu.edu.cn (J.L.); 23115198@bjtu.edu.cn (C.M.); 22110515@bjtu.edu.cn (Y.Z.); wangxiao20011101@126.com (X.W.)

**Keywords:** heterodyne interferometric sensing, nonlinear error, PINN, self-supervised learning, intelligent signal processing, industrial sensors, precision metrology sensors

## Abstract

**Highlights:**

**What are the main findings?**

- We proposed a self-supervised learning paradigm that leverages frequency-domain priors to generate high-confidence pseudo-labels for identifying nonlinear errors in heterodyne interferometric sensing systems.
- We developed a Physics-Informed Neural Network (PINN) featuring a differentiable physical layer to impose explicit hard constraints on the architecture, enabling precise identification of nonlinear physical properties against high background noise.

**What are the implications of the main findings?**

- The method enables high-precision calibration of nonlinear errors in heterodyne interferometric sensing systems without requiring ground-truth labels or complex hardware modifications.
- It ensures the physical consistency of identification results and enhances the robustness of the identification process against high background noise by integrating explicit physical hard constraints.

**Abstract:**

Although laser heterodyne interferometric sensing systems offer exceptional theoretical resolution, practical precision is constrained by intractable nonlinear errors stemming from optical imperfections. Conventional compensation methods suffer from hardware dependency, complexity, and performance degradation under low signal-to-noise ratios (SNR). To address this, we propose a precision calibration method using a self-supervised Physics-Informed Neural Network (PINN) guided by frequency-domain priors with harmonic distribution characteristics. This approach establishes a robust compensation model by inverting equivalent parameter sets and error curves in a single step. Specifically, leveraging high-precision displacement references, the method extracts measurement residuals containing periodic physical features. Subsequently, it integrates frequency-domain priors into a physically constrained network architecture: theoretical frequency characteristics construct masks to generate high-confidence pseudo-labels, while the error equation is recast as a differentiable physical layer imposing explicit hard constraints during forward propagation. This mechanism enables precise identification of the system’s nonlinear physical properties against high background noise. Experimental results show that the root-mean-square (RMS) value of the nonlinear error was reduced from 1.90 nm to 0.23 nm, with a compensation rate reaching up to 88.13%. This method provides a reliable framework for the intelligent calibration and error self-characterization of heterodyne interferometric industrial sensors in the field of precision metrology sensors.

## 1. Introduction

Laser heterodyne interferometric sensing systems, characterized by their high resolution, large measurement range, and robust environmental adaptability, have established themselves as the benchmark optical sensing equipment in the fields of ultra-precision manufacturing and metrology [1]. Consequently, they are extensively employed in cutting-edge applications such as lithography scanners and ultra-precision machine tools [2,3,4,5]. As semiconductor manufacturing processes advance toward the 7 nm node and beyond, the industry imposes increasingly stringent demands for real-time measurement accuracy, necessitating precision at the nanometer or even atomic scale [6].

However, arising from factors such as the non-ideal characteristics of polarizing beam splitters (PBS), waveplate installation errors, frequency mixing in the laser source, and optical ghost reflections [7], periodic nonlinear errors are inevitably introduced into the interference sensing signals [8,9]. Typically ranging from several to over ten nanometers, these errors degrade signal fidelity and serve as a critical bottleneck restricting accuracy improvements in nanometrology sensing systems, thereby emerging as a focal point of research for scholars in the field [10,11].

To mitigate this nonlinear error, researchers initially conducted a series of investigations from a hardware perspective. Lu et al. established a model based on the amplitude fluctuation characteristics of the beat frequency signal, achieving precise decoupling and identification of laser polarization non-orthogonality and PBS transmission coefficients. However, this method necessitates the insertion of a rotatable analyzer into the optical path, making it difficult to realize in situ correction of time-varying errors in practical operational environments [12]. Guo et al. proposed a dual-detector heterodyne interference optical path, which suppressed nonlinear errors by more than 30 times while simultaneously doubling the measurement resolution. However, this scheme necessitates additional optical components and modifications to the interferometer structure, thereby increasing system complexity and alignment difficulty [13]. Straube et al. successfully reduced the nonlinear error to 0.22 nm through a complex spatially separated optical path design. However, this method necessitates the construction of intricate optical paths and the use of expensive precision optical components [14]. Lawall et al. constructed a dual-frequency heterodyne interference system using acousto-optic modulators (AOMs). By strictly isolating the reference and measurement beams in space, they successfully reduced the periodic nonlinear error to the 10 pm level, achieving an accuracy improvement of two orders of magnitude compared to traditional commercial instruments. However, the common drawbacks of such schemes lie in their complex optical designs, large physical footprints, and high costs, coupled with extremely high requirements for environmental stability [15]. Consequently, these hardware-centric modifications are largely unsuitable for cost-sensitive industrial environments and highly integrated precision sensing systems.

In comparison, signal processing algorithms for error compensation have attracted increasing attention due to their cost-effectiveness and flexibility. In 1981, Heydemann proposed a correction model based on the least squares method, successfully addressing fundamental error issues such as gain mismatch and phase non-orthogonality in interference signals [16]. Wu et al. further refined the nonlinear error compensation scheme based on ellipse fitting, achieving significant accuracy improvements in homodyne interferometry [17]. Although theoretically applicable to heterodyne interferometry, this method is limited by its dependence on complete signal periods. Xie et al. proposed an iterative compensation algorithm based on Field-Programmable Gate Array (FPGA) digital phase-locked loop (DPLL) technology (Iterative DPLL). This method successfully suppressed the nonlinear error to within 0.8 nm. However, it relies on high-cost dedicated hardware (such as FPGAs and high-speed Analog-to-Digital Converters (ADCs)), and its robustness under low SNR conditions remains to be verified [18]. Additionally, Bridges et al. proposed a dual-wavelength interferometry scheme, successfully reducing the residual nonlinear error from 84 pm to 11 pm. However, this method significantly increases hardware complexity and cost and relies primarily on offline post-processing algorithms [19]. Li et al. proposed an error compensation strategy based on post-demodulation data. By performing segmentation and statistical averaging on the measurement signal to extract error features, they successfully achieved nonlinear correction without the need for original quadrature signals. However, constrained by its statistical reliance on periodic segmentation and the assumption of steady-state errors, this method struggles to cope with sensor signal distortions induced by non-periodic motion and strong noise [20].

Intelligent signal processing methods based on deep learning have been widely adopted across numerous scientific and industrial fields [21,22,23,24,25,26]. Due to its exceptional nonlinear mapping and feature extraction capabilities, it is extensively employed to address complex nonlinear problems [27,28,29,30]. Heo et al. utilized a Deep Frequency Neural Network (DFNN) to achieve multi-error fitting in heterodyne interferometry. However, this method relies on ground truth labels provided by high-precision capacitive sensors, as well as a hardware architecture based on dual laser heads [31]. Olyaee et al. proposed employing a Self-supervised Generative Neural Network (SGNN) to fit nonlinear error models in heterodyne interferometry, achieving picometer-level compensation accuracy under ideal simulation conditions [32]. However, this method lacks verification under realistic noise conditions. Fundamentally, these two methods merely achieve numerical fitting between inputs and outputs. Due to the absence of explicit constraints from underlying physical sensing mechanisms, the models are highly susceptible to overfitting and generalization failure when encountering parameter drift or strong noise interference in practical operating conditions.

In summary, conventional nonlinear error compensation schemes are predominantly hardware-dependent, suffering from limitations such as high system complexity and elevated costs. Early neural network-based approaches function as purely numerical fitting models that rely on ground truth labels and fail to adapt to the complex, low SNR conditions inherent in realistic industrial environments. Building upon existing research on nonlinear error compensation, this paper proposes a nonlinear error parameter identification and precision calibration and compensation method for heterodyne interferometric sensing systems based on a self-supervised PINN guided by frequency-domain priors. This approach establishes a physics-driven training paradigm that eliminates the dependency on ground truth labels. The feasibility of the proposed method is validated through experiments designed under measured strong noise backgrounds.

## 2. Nonlinear Error in Heterodyne Interferometric Sensing Systems

**Figure 1:**
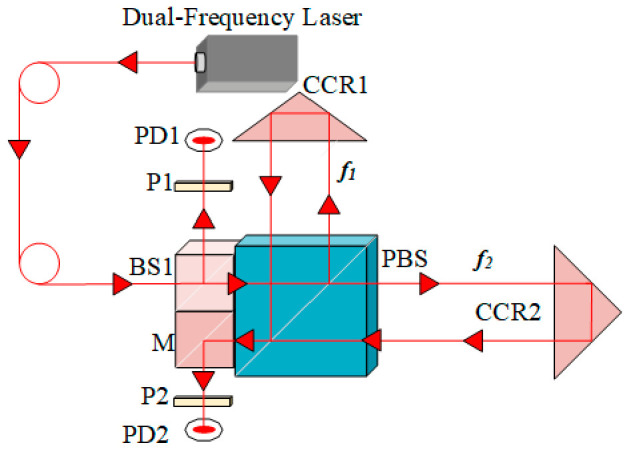
Schematic diagram of the basic optical path of the heterodyne interferometric sensing system.

Figure 1 illustrates the schematic optical configuration of the heterodyne interferometric sensing system employed in this study. Key components include a dual-frequency laser source, a beam splitter (BS1), polarizers (P1 and P2), photodiodes (PD1 and PD2), a polarizing beam splitter (PBS), a mirror (M), and corner cube retroreflectors (CCR1 and CCR2). Polarizers P1 and P2 are both oriented at 45° to obtain the optimal interference signal. Let *f*_1_ denote the frequency of the optical component reflected by the PBS, and *f*_2_ denote the frequency of the transmitted component.

In practical scenarios, however, ideal conditions are rarely met due to imperfections in optical alignment, manufacturing processes, and component quality. Consequently, the laser beams entering the arms of the heterodyne interferometric sensing system deviate from theoretically pure single-frequency states; instead, they inevitably suffer from frequency mixing (or leakage), where the orthogonal frequency component is introduced to a certain extent. Therefore, the optical fields propagating within the two interferometer arms can be mathematically expressed as follows [33]:

(1) $$\begin{array}{l} E_{1}=a\sin(2\pi f_{1}t+\varphi_{10}+\varphi_{1})+b\sin(2\pi f_{1}t+\varphi_{10}+\varphi_{2}-\varepsilon )\\ E_{2}=c\sin(2\pi f_{2}t+\varphi_{20}+\varphi_{2})+d\sin(2\pi f_{2}t+\varphi_{20}+\varphi_{1}+\sigma ) \end{array},$$

In Equation (1), *a*, *b*, *c*, and *d* represent the optical amplitudes; *φ*_10_ and *φ*_20_ denote the initial phases of the reference and measurement beams, respectively; *ε* and *σ* indicate the initial additional phase shifts; and *φ*_1_ and *φ*_2_ correspond to the phase shifts induced as light propagates through the two interferometer arms. Consequently, the formula for calculating the nonlinear error can be derived as follows:

(2) $$\begin{array}{l} \rho =\arctan\left[\frac{b\sin(\Delta \varphi +\varepsilon )}{a+b\cos(\Delta \varphi +\varepsilon )}\right]\\ \eta =\arctan\left[\frac{d\sin(\Delta \varphi +\sigma )}{c+d\cos(\Delta \varphi +\sigma )}\right]\\ \gamma =\rho +\eta \end{array},$$

According to Equation (2), it can be inferred that frequency mixing within the interference optical path introduces an additional phase error, *γ*, into the beat frequency interference signal. This error varies periodically with the measured phase difference Δ*φ*, resulting in a nonlinear relationship between the detected phase displacement Δ*φ* and the measured length Δ*l*. This relationship is expressed as follows:

(3) $$\Delta l=\frac{\lambda }{4\pi }(\Delta \varphi +\gamma )$$

where *λ* denotes the laser wavelength. Evidently, in heterodyne interferometric sensing systems, if the values of a, b, c, and d, as well as ε and σ, can be determined (i.e., the nonlinear error curve formula), the numerical value of the nonlinear error γ can be derived by combining them with the phase displacement Δ*φ*, thereby achieving error compensation.

## 3. Physics-Informed Neural Network Compensation Method Guided by Frequency-Domain Priors

To address the problem of nonlinear error compensation in laser heterodyne interferometry, this paper proposes a precise calibration and parameter identification method for heterodyne interferometric sensing systems. This method is based on a self-supervised Physics-Informed Neural Network (PINN) guided by frequency-domain priors. By recasting Equation (2) from the preceding section, the transformed expression can be derived as:

(4) $$\begin{array}{l} \rho =\arctan\left[\frac{\frac{b}{a}\sin(\Delta \varphi +\varepsilon )}{1+\frac{b}{a}\cos(\Delta \varphi +\varepsilon )}\right]\\ \eta =\arctan\left[\frac{\frac{d}{c}\sin(\Delta \varphi +\sigma )}{1+\frac{d}{c}\cos(\Delta \varphi +\sigma )}\right] \end{array}$$

Let *k*_1_ and *k*_2_ denote the mixing ratios in the two optical paths:

(5) $$k_{1}=\frac{b}{a},\;k_{2}=\frac{d}{c}$$

The mixing ratio *k*, along with the initial additional phase shifts *ε* and *σ*, are critical parameters for constructing the nonlinear error curve. Based on this, a PINN compensation system guided by frequency-domain priors is constructed. Through the deep coupling of data-driven approaches and physical constraints, this system establishes a closed-loop iterative optimization process. Ultimately, it identifies the nonlinear error parameters and curves of the heterodyne interferometric sensing systems, thereby achieving error compensation in subsequent measurements.

**Figure 2:**
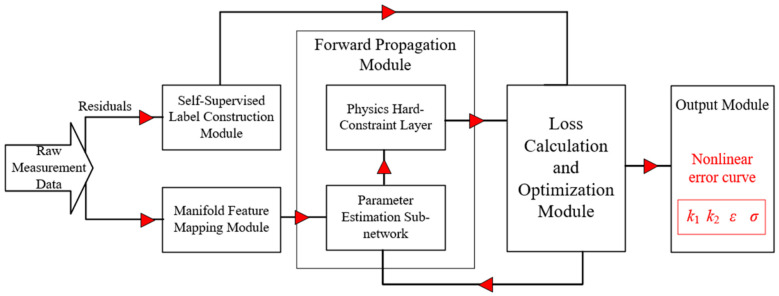
Schematic diagram of the logical principle for the nonlinear error compensation method in heterodyne interferometry based on a self-supervised Physics-Informed Neural Network guided by frequency-domain priors.

The schematic diagram of the design principle is shown in Figure 2. The system input stage employs a manifold mapping strategy, mapping scalar displacement data into high-dimensional sine-cosine feature vectors. Simultaneously, the residual between the heterodyne measurement value and the true displacement value is obtained and processed via the system’s built-in frequency-domain prior technique, thereby generating self-supervised pseudo-labels. In the inference pathway, the physical parameters predicted by the network are passed through a hard physical constraint layer, which converts the abstract parameters into predicted waveforms adhering to physical laws. Guided by the gradients of a composite loss function, the model converges to a physically consistent solution through iterative optimization. Ultimately, it overcomes broadband noise interference, achieving high-precision identification of nonlinear error parameters and reconstruction of the nonlinear error curve.

### 3.1. Manifold Feature Mapping for Sensing Signals

In the signal processing stage, if raw measurement data are directly used as inputs, the continuous increase in phase values obscures the periodic patterns of nonlinear errors, making it difficult for the network to capture repetitive features over large displacement ranges. Simply employing the modulo operation for phase wrapping results in abrupt numerical discontinuities at the cycle boundaries. Such discontinuities cause gradient calculation failures or severe oscillations during backpropagation. This prevents the model from converging to smooth error curves that adhere to physical laws, ultimately leading to compensation failure.

**Figure 3:**
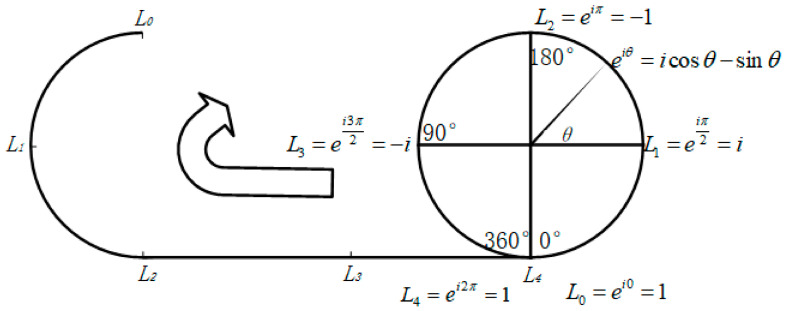
Schematic diagram of the principle of manifold feature mapping.

To address this issue, instead of directly using the linearly increasing displacement *L* as the input, the network constructs a continuous periodic feature space. The basic principle of this approach is illustrated in Figure 3. According to the theory of nonlinear errors in heterodyne interferometry, the magnitudes of the nonlinear errors corresponding to positions *L*_0_ and *L*_4_ are identical.

Therefore, let *L_meas_* (*t*) denote the measured displacement at time *t*. Then:

(6) $$\phi \left(t\right)=L_{meas}\left(t\right)\cdot \frac{4\pi }{\lambda }$$

In Equation (6), *λ* denotes the laser wavelength. Subsequently, the input vector *x*(*t*) is constructed as follows:

(7) $$x\left(t\right)=\left[\begin{array}{c} \sin\left(\phi \left(t\right)\right)\\ \cos\left(\phi \left(t\right)\right) \end{array}\right]$$

Mapping the one-dimensional linearly increasing measurement phase space onto a high-dimensional trigonometric manifold space resolves the discontinuity issues caused by phase wrapping. This enables the network to perceive the periodic fluctuation patterns of the sensing system’s nonlinear errors within a continuous and closed feature space.

### 3.2. Frequency-Domain Prior Label Reconstruction Methods

In actual measurement scenarios, directly using noisy residuals as labels makes it difficult for the network to converge due to strong noise interference. To address this, leveraging the frequency-domain sparsity and physical homology of nonlinear errors, a pseudo-label construction mechanism based on frequency-domain priors is developed to resolve the supervision challenge under low SNR conditions.

According to the theory of multi-beam interference mixing, the nonlinear error *e_nl_*(*x*) can be approximated as a linear combination of the first- and second-order harmonics [34]:

(8) $$e_{nl}\left(x\right)\approx A_{1}\sin\left(2\pi v_{1}x+\varphi_{1}\right)+A_{2}\sin\left(2\pi v_{2}x+\varphi_{2}\right)$$

In Equation (8), *x* denotes the measured displacement, *υ*_1_ = 2/*λ* and *υ*_2_ = 4/*λ* represent the fundamental spatial frequency and the second-harmonic frequency, respectively. This physical characteristic indicates that the energy of the target signal is highly concentrated at these two specific frequency points, *υ*_1_ and *υ*_2_. However, since the coefficient of the first-order term is significantly larger than that of the second-order term (*A*_1_ >> *A*_2_), the second-order features are easily masked by noise under low SNR conditions, making their extraction difficult. Therefore, under low SNR conditions, priority should be given to locking onto the high-energy first-order features to ensure robustness.

To accurately extract error features, the residual sequence is first mapped to the frequency domain using the Discrete Fourier Transform (DFT). Unlike traditional data-driven filtering methods, this study leverages physical priors to directly lock onto target frequencies in the frequency domain. Given the total length of a measurement input segment as *L_scan_*, the theoretical fundamental wave index *K*_1_ can be obtained:

(9) $$K_{1}=round\left(\frac{L_{scan}}{\lambda /2}\right)$$

Similarly, the second-harmonic index is given by *K*_2_ = 2*K*_1_. Based on the aforementioned feature locking mechanism, a frequency-domain band-pass mask matrix *M* is constructed. To account for spectral leakage effects caused by finite sampling, the mask retains a passband window with a bandwidth of *ω* around the theoretical locking frequency points. Simultaneously, hard threshold truncation is applied to coefficients in non-physical frequency bands containing low-frequency thermal drift and high-frequency electronic noise, forcing them to zero. Consequently, the cleaned spectrum is obtained. Finally, the Inverse Discrete Fourier Transform (IDFT) is employed to map the cleaned spectrum back to the time domain, thereby yielding the constructed pseudo-label *r_refined_*. The core function of this module lies in leveraging prior physical knowledge to achieve signal energy focusing and noise energy blocking. Theoretically, the SNR gain resulting from this operation can be approximated as the ratio of the total bandwidth to the retained bandwidth:

(10) $$G_{SNR}\approx 10\log_{10}\left(\frac{N}{2\omega }\right)$$

where *N* denotes the number of samples and *ω* represents the bandwidth. For a typical configuration of *N* = 3000 and *ω* = 8, this module yields an SNR improvement of approximately 22.73 dB (or approximately 25.74 dB if only the first-order term is retained). This provides a clear and convergent optimization gradient path for the PINN under severe noise conditions, fundamentally resolving the gradient masking problem.

### 3.3. Construction of Differentiable Physical Hard Constraints and Forward Evolution Mechanism

This module serves as the core component for achieving the deep integration of physical mechanisms and data-driven approaches. To ensure robust parameter identification and waveform reconstruction under severe noise conditions, an architecture characterized by “parameter estimation followed by physical constraints” is constructed.

The front end employs a lightweight fully connected neural network as a parameter estimator to establish a nonlinear mapping from the manifold features of the input signal to the physical parameter space. To ensure that the output parameters adhere to the objective physical laws of heterodyne interferometry, a physics-aware asymmetric activation mechanism is introduced at the network output layer: The Sigmoid function is utilized to strictly constrain the mixing coefficients within a reasonable physical interval, thereby preventing parameter overfitting and physical distortion induced by noise. Simultaneously, the linear output characteristics of the phase parameters are maintained to accommodate full-cycle phase drifts.

The core lies in the backend physical hard constraint layer. This layer is a fixed, differentiable mathematical operator, whose transfer function strictly corresponds to the analytical equation of nonlinear errors in heterodyne interferometry. This layer receives the parameter estimates *θ* from the parameter estimation sub-network and the raw measurement phase Δ*φ*, performing a deterministic forward calculation. The output predicted error waveform *e_nl_* is defined as follows:

(11) $$e_{nl}\left(\Delta \varphi ;\theta \right)=P\left(\Delta \varphi ,k_{1},k_{2},\varepsilon ,\sigma \right)$$

The specific computational logic is expanded as follows:

(12) $$e_{nl}=\arctan\left[\frac{k_{1}\sin(\Delta \varphi +\varepsilon )}{1+k_{1}\cos(\Delta \varphi +\varepsilon )}\right]+\arctan\left[\frac{k_{2}\sin(\Delta \varphi +\sigma )}{1+k_{2}\cos(\Delta \varphi +\sigma )}\right]$$

Another key characteristic of this layer is its differentiability. During backpropagation, the gradients of the loss function with respect to the parameters *θ* can be precisely calculated via the chain rule. For example, the partial derivative with respect to the mixing coefficient *k*_1_ is given by the following:

(13) $$\frac{\partial e_{nl}}{\partial k_{1}}=\frac{\sin\left(\Delta \varphi +\varepsilon \right)}{1+2k_{1}\cos\left(\Delta \varphi +\varepsilon \right)+k_{1}^{2}}$$

This implies that the presence of the physical layer not only regulates the output waveform but also provides the optimizer with a gradient descent path of clear physical significance.

In this module, the network is constrained to focus on extracting periodic error components that conform to physical laws, thereby theoretically achieving the automatic decoupling of nonlinear errors from random noise. This ensures high robustness and parameter identification accuracy of the algorithm under severe noise interference.

### 3.4. Adaptive Loss and Optimization

**Figure 4:**
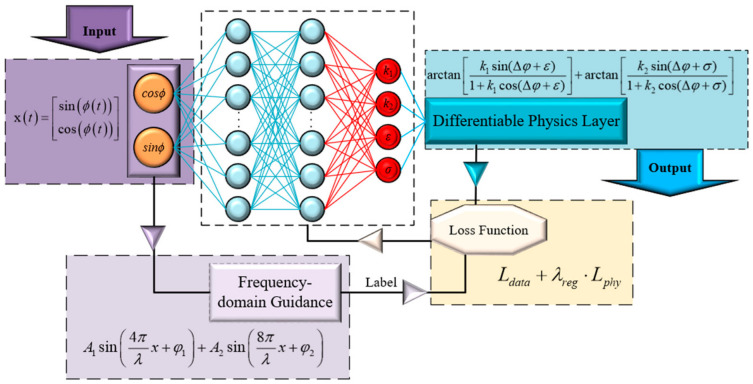
Architecture of the Frequency-domain-guided Physics-Informed Neural Network.

Figure 4 illustrates the overall architecture of the frequency-domain guided PINN. Finally, addressing the drastic variation of the SNR with distance in heterodyne interferometry, traditional loss functions with fixed weights struggle to balance performance across the entire measurement range. Consequently, this module formulates a composite objective function incorporating the waveform reconstruction error *L_data_* and the physical prior regularization *L_phy_*:

(14) $$L_{total}=L_{data}+\lambda_{reg}\cdot L_{phy}$$

where:

(15) $$L_{data}\left(\theta \right)=\frac{1}{N}{\sum_{i=1}^{N}\left\|e_{nl}\left(\Delta \varphi_{i};\theta \right)-r_{refined}\left[i\right]\right\|}^{2}$$

(16) $$L_{phy}\left(\theta \right)=\sum_{j=1}^{2}{\left(k_{j}-k_{prior}\right)}^{2}$$

In the formula, *e_nl_* represents the predicted error waveform, *r_refined_* is the pseudo-label, N denotes the number of sampling points, and *θ* is the set of physical parameters. Additionally, *k_prior_* is a preset empirical value based on the hardware specifications of the optical system, serving to prevent parameters from deviating from a reasonable physical range. *λ_reg_* serves as the regularization weight hyperparameter, which determines the influence of physical priors during the optimization process. To balance the weights of data-driven components and physical constraints, this coefficient adopts a piecewise adaptive strategy based on the SNR:

(17) $$\lambda_{reg}=\left\{\begin{array}{c} 0.5,\left(Low-SNR\right)\\ 0.05,\left(High-SNR\right) \end{array}\right.$$

In high SNR regions, a smaller *λ_reg_* is adopted to allow the network to capture subtle individual variations, achieving high-precision fitting. Conversely, in low SNR regions, a larger *λ_reg_* is employed to suppress noise gradients and force the solution to converge to a physically feasible region, thereby realizing high-robustness estimation.

## 4. Experiments and Analysis

**Figure 5:**
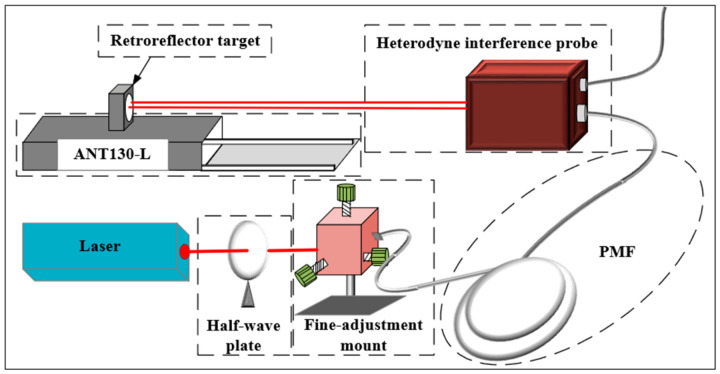
Schematic illustration of the experimental measurement and sensing system.

**Figure 6:**
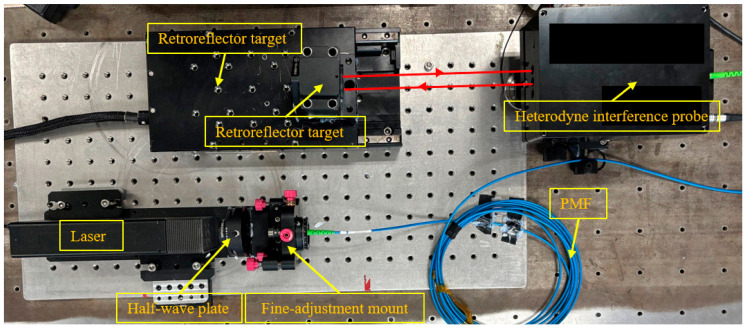
Physical photograph of the experimental measurement and sensing system.

The experimental setup is illustrated in Figure 5 and Figure 6, which consists of a dual-frequency laser, a half-wave plate, an adjustment mount, a Polarization-Maintaining Fiber (PMF), an interferometer head, a target mirror, and a high-precision AEROTECH nano-positioning stage (model ANT130-L).

To address the objective challenge of obtaining ground truth for sub-nanometer nonlinear errors under constrained laboratory conditions, this paper establishes an experimental verification framework based on measured noise fingerprints. This method constructs a hybrid dataset combining mathematical precision and physical realism by coupling a theoretical error model consistent with the interference mechanism into environmental background noise and circuit noise data collected from real instruments. This strategy not only resolves the challenge of comparing nonlinear error ground truth curves in the final experiment but also fully preserves the spectral characteristics and stochastic statistical properties of the actual measurement environment.

### 4.1. Data Acquisition and Construction of Noise Fingerprint Library

To ensure that the experiment accurately reproduces the stochastic interference characteristics of the actual measurement site, a noise fingerprint library based on empirical sampling was first established. The experimental data were derived from an open heterodyne interferometric sensing system, as illustrated in Figure 6. Fixed-point sampling was conducted at two positions (10 mm and 100 mm), with 100 samples collected for each.

**Figure 7:**
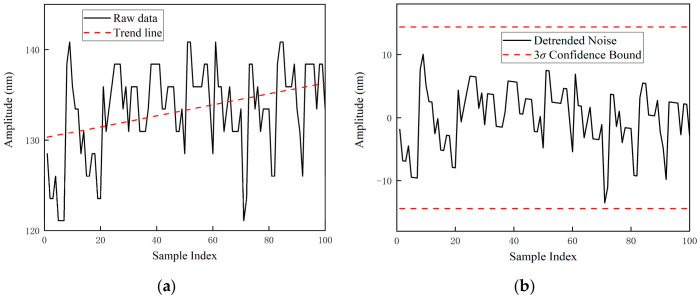
Signal Preprocessing and Noise Cleaning Results at the 10 mm Measurement Position. (**a**) Raw Detrended Fluctuation Signals; (**b**) Valid Noise Sequences Extracted via the 3*σ* Criterion.

**Figure 8:**
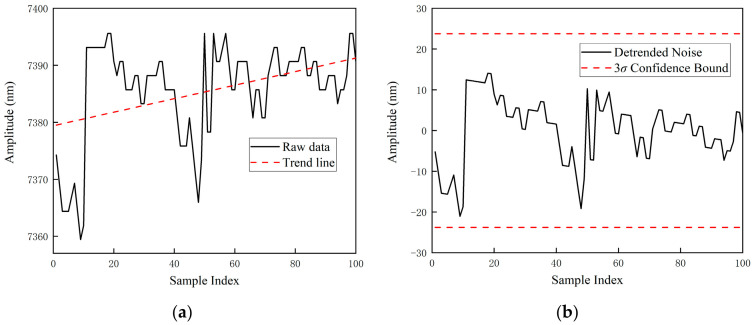
Signal Preprocessing and Noise Cleaning Results at the 100 mm Measurement Position. (**a**) Raw Detrended Fluctuation Signals; (**b**) Valid Noise Sequences Extracted via the 3*σ* Criterion.

To address low-frequency non-stationary trends induced by slow ambient temperature drifts and laser thermal effects, a linear regression detrending algorithm is first employed for elimination, thereby isolating high-frequency random fluctuation components. Subsequently, to mitigate sporadic impulsive interference from the circuit, a statistical filter based on the 3σ criterion is applied to these detrended residuals. Any sample points deviating from the mean by more than three standard deviations are identified as non-physical outliers and subsequently discarded. The data processing results for the two measurement positions are detailed in Figure 7a,b and Figure 8a,b, respectively.

### 4.2. High-Fidelity Reconstruction of Heterodyne Sensing Signals Under Non-Ideal Conditions

The input signal is constructed based on the physical mechanism of heterodyne interference, with the linear scanning input range set to 0–20 µm and the number of sampling points set to 3000. By substituting a set of typical parameters (*k*_1_ = 0.035, *k*_2_ = 0.025, *ε* = 45°, *σ* = −10°) into Equations (4) and (5), the theoretical error curve is generated. To address the data magnitude mismatch between the static samples (100) and the dynamic scanning process (3000), this paper employs a random resampling technique with replacement. This approach augments the short-sequence measured noise cleaned in Section 4.1 into a long-sequence background noise that matches the constructed input steps.

The construction of input data aims to reproduce the raw measurement state of the sensor under non-ideal operating conditions. To simulate realistic interferometer readings, the network input signal is synthesized by superimposing the long-sequence background noise and the theoretical nonlinear error curve onto an ideal linear displacement scan. This approach fully preserves the realistic environmental noise characteristics across the entire frequency band, as well as the nonlinear errors. The experiment constructs a feature tensor X based on orthogonal trigonometric functions:

(18) $$X={\left[\sin\left(\frac{4\pi }{\lambda }Input\right),\cos\left(\frac{4\pi }{\lambda }Input\right)\right]}^{T}$$

**Figure 9:**
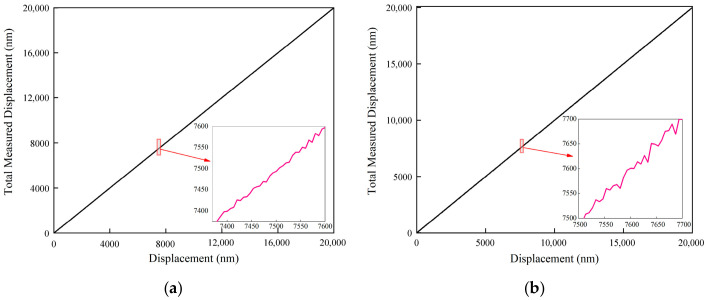
Measurement input signals constructed based on background noise at different positions: (**a**) 10 mm position; (**b**) 100 mm position.

The inputs constructed based on the background noise at the 10 mm and 100 mm positions are illustrated in Figure 9a,b.

### 4.3. Validation of the Effectiveness of the Physics-Driven Reconstruction Mechanism

#### 4.3.1. Reconstruction Fidelity of Frequency-Domain Prior Pseudo-Labels Under Strong Noise Backgrounds

**Figure 10:**
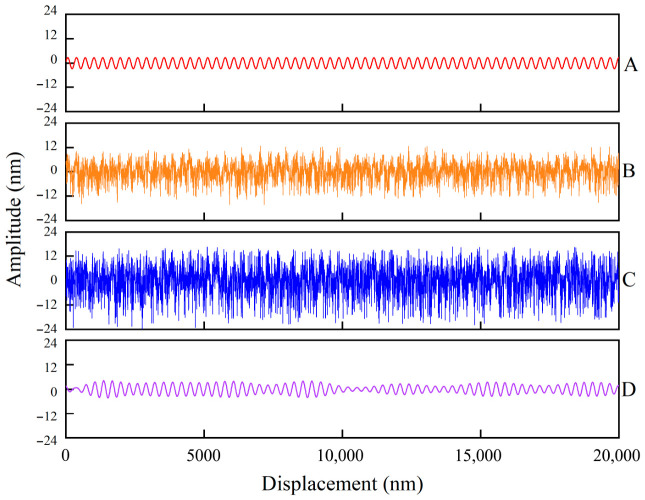
Illustration of the training data generation process and pseudo-ground truth label construction. (**A**). Ground truth of the nonlinear error; (**B**). Training data superimposed with measured noise at 10 mm; (**C**). Training data superimposed with measured noise at 100 mm; (**D**). Frequency-domain-guided pseudo-ground truth (100 mm noise).

The input data were fed into the PINN, and the intermediate output states of the frequency-domain prior module were monitored. Figure 10 illustrates the complete process of pseudo-label reconstruction. Figure 10A presents the theoretical error baseline curve defined in Section 4.2, which also serves as the ground truth for benchmarking the network’s prediction results. Figure 10B,C display the residuals (original labels) after decoupling the true displacement values, constructed based on the background noise at the 10 mm and 100 mm positions, respectively. When the SNR is low (experimentally set to SNR < −10 dB), the labels require processing via frequency-domain priors. Figure 10D illustrates the pseudo-labels generated after frequency-domain prior processing of the high-noise samples based on the background noise at the 100 mm position.

Experimental results demonstrate that even though the source data in Figure 10C is severely corrupted by strong noise, the pseudo-labels reconstructed via the frequency-domain prior still accurately recover the periodicity and amplitude characteristics of the ground truth. This result confirms that the strategy successfully achieves the concentration of signal energy and the suppression of noise energy, providing high-confidence convergence targets for the subsequent PINN and ensuring the trainability of deep learning in low SNR environments.

#### 4.3.2. Nonlinear Error Waveform Reconstruction and Verification of Fitting Consistency

**Figure 11:**
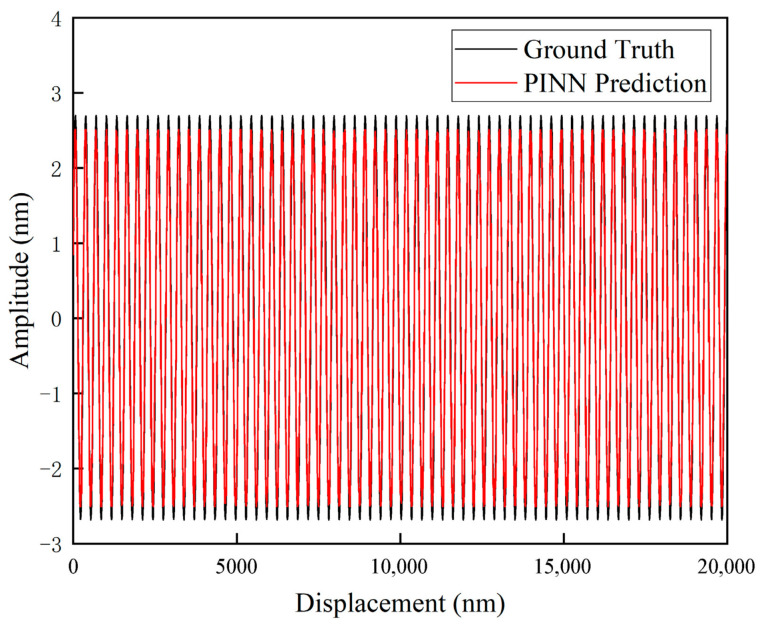
Comparison Between Neural Network Predicted Results and Ground Truth Based on Background Noise at the 100 mm Position.

**Figure 12:**
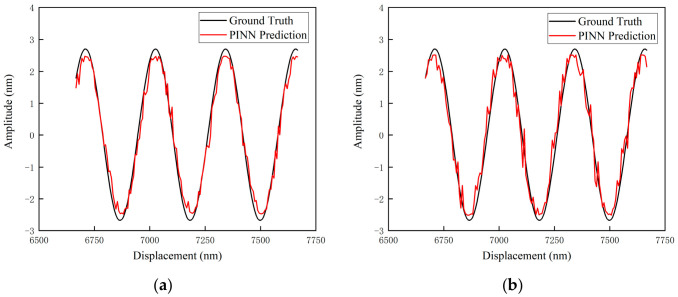
Local illustration of the comparison between neural network predicted results and ground truth based on background noise at different positions: (**a**) 10 mm position; (**b**) 100 mm position.

The final output curves of the network were obtained. Figure 11 presents the comparison between the neural network predictions and the ground truth, based on the background noise at the 100 mm position. Figure 12a,b illustrate the local comparisons between the predicted values and the ground truth, corresponding to the background noise at the 10 mm and 100 mm positions, respectively.

The physical nature of the nonlinear error in heterodyne interferometers is a periodic function composed of first- and second-order harmonic components. Therefore, under ideal noise-free conditions, its evolution curve should exhibit a smooth quasi-sinusoidal waveform. However, in Figure 11 and Figure 12, the output prediction curves exhibit jitter. This phenomenon does not imply that the PINN model failed to capture the nonlinear laws; rather, it is determined by the stochastic characteristics of the input data.

This phenomenon is attributed to the point-wise mapping mechanism adopted by the PINN architecture. Environmental micro-vibrations and detector noise at the input are inevitably superimposed onto the measured phase and are subsequently propagated to the output through the physical layer equations, governed by the law of error propagation. This phenomenon demonstrates that the PINN network is capable of faithfully reflecting the true physical response of nonlinear errors varying with the instantaneous phase under noisy measurement conditions.

### 4.4. Comprehensive Performance Evaluation and Comparative Study of Models

To quantitatively evaluate the degree of reconstruction of the nonlinear error curve output by the PINN network with respect to the theoretical ground truth curve, the Pearson correlation coefficient between the two was calculated. This coefficient reflects the strength of the linear correlation between two variables, i.e., the synchronization in their variation trends.

(19) $$r=\frac{\sum_{i=1}^{N}\left(x_{i}-x_{j}\right)\left(y_{i}-y_{j}\right)}{\sqrt{\sum_{i=1}^{N}{\left(x_{i}-x_{j}\right)}^{2}\sqrt{\sum_{i=1}^{N}{\left(y_{i}-y_{j}\right)}^{2}}}}$$

where *r* is the Pearson correlation coefficient, and *N* is the total number of sampling points along a single scanning path; *x_i_* and *y_i_* represent the theoretical ground truth of the nonlinear error and the network predicted value at the *i* sampling point, respectively; and *x_j_* and *y_j_* denote the arithmetic means of the two sequences.

In the experiments constructed based on measured background noise at the 10 mm and 100 mm positions, the calculated Pearson correlation coefficients and nonlinear error compensation rates between the nonlinear error curves output by the PINN network and the ground truth curves are presented in Table 1.

Table 1 indicates that the proposed system exhibits excellent robustness under strong noise environments. The correlation coefficient between the neural network prediction curve and the nonlinear error ground truth curve reaches as high as 0.9939. Even under low SNR conditions, the network remains capable of accurately capturing the phase characteristics of the nonlinear error, achieving a correlation coefficient of 0.9817. A comparison of the RMS values before and after compensation reveals that the proposed method reduces the system’s measurement uncertainty from 1.9 nm to within 0.37 nm, achieving an error compensation rate exceeding 88%.

#### 4.4.1. Ablation Analysis of Key Physical Mechanisms

**Figure 13:**
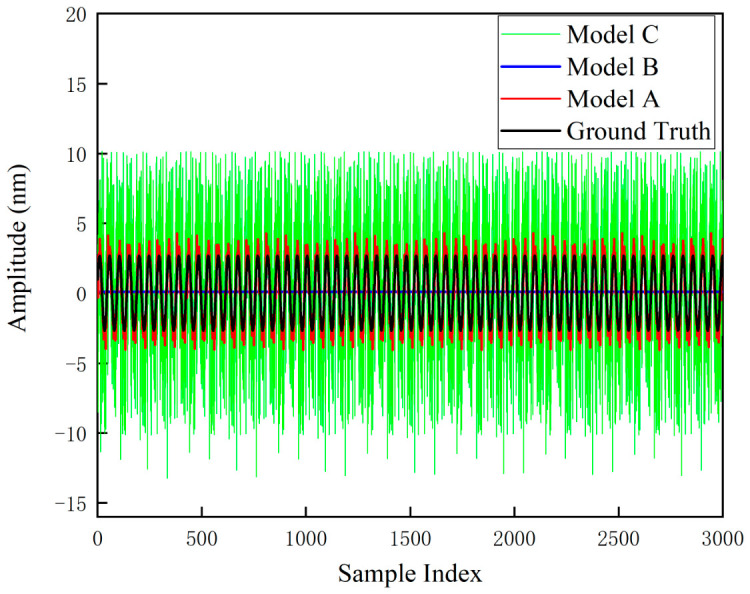
Comparison of nonlinear error curves generated by the three models.

**Figure 14:**
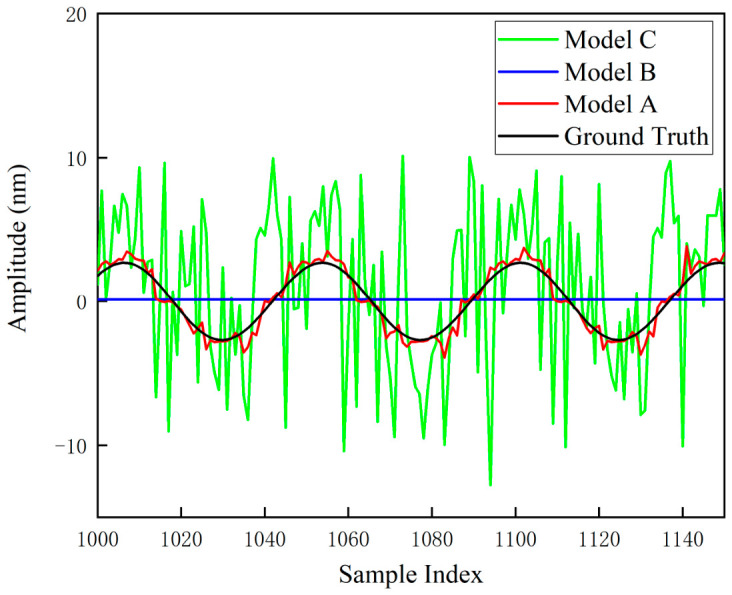
Zoomed-in comparison of the nonlinear error curves.

To validate the robustness of the proposed architecture under extreme operating conditions, a sparse data ablation experiment was designed to simulate the measurement state of heterodyne interferometry under high-speed motion of the measured object. The training data were down-sampled to 5% of the total volume (150 points), and real strong background noise was introduced to compare three neural network models. The experimental results are shown in Figure 13, while Figure 14 displays the locally magnified details of Figure 13.

Model C: This model retains the physical hard constraint layer and directly utilizes noisy sparse samples as training targets. Benefiting from the constraints of the physical equations, the output maintains a basic sinusoidal waveform. However, strong background noise severely interferes with the identification of physical parameters, resulting in significant systematic deviations across the entire range.

Model B: This model eliminates the physical hard constraint layer, representing a purely data-driven DNN architecture. Experiments demonstrate that the model exhibits extremely high sensitivity to hyperparameters and instability under sparse data conditions. Although adjusting the network capacity occasionally yields favorable results, the model is prone to significant fluctuations between overfitting and mode collapse. This high degree of uncertainty renders it difficult to meet the robustness requirements of practical measurements.

Model A: As the novel PINN architecture proposed in this paper, Model A exhibits superior generalization capability. The physical layer strictly constrains the solution space within the theoretical manifold, fundamentally eliminating the risks of overfitting and oscillation observed in Model B; meanwhile, the frequency-domain prior effectively corrects the parameter drift induced by noise, resolving the bias issue associated with Model C. The synergistic effect of these two mechanisms enables the model to achieve high-precision and high-robustness error reconstruction, even under extreme conditions with only 5% of the data.

The results of the comparison between the outputs of the three models and the nonlinear error ground truth curve are presented in Table 2.

#### 4.4.2. Performance Benchmarking Against Existing Mainstream Compensation Methods

Nonlinear error compensation technology has primarily evolved from hardware suppression to software compensation. Early hardware-based solutions suppressed nonlinear errors at the physical level, achieving satisfactory compensation results in laboratory settings. However, these methods are characterized by prohibitively high hardware costs and complex optical path structures, often necessitating customized laser sources or precision optical isolation components. These factors render them difficult to deploy in cost-sensitive industrial fields characterized by harsh environments. Currently, compensation techniques centered on signal processing algorithms have emerged as a research hotspot. Table 3 presents a detailed comparison of the performance metrics between the proposed scheme and representative methods in the field.

Although traditional signal processing algorithms, represented by iterative DPLL, have lowered hardware barriers and improved compensation accuracy, their precision is constrained by the algorithmic models, reaching only the 0.80 nm level. Furthermore, in the face of strong noise interference common in industrial settings, their anti-noise robustness is mediocre, making it difficult to meet the requirements of ultra-precision measurement. Meanwhile, existing deep learning methods suffer from the drawbacks of dependence on hardware and ground truth labels. While the DFNN method incorporates neural networks, it fundamentally relies on expensive dual-optical path systems and ground truth labels, achieving an accuracy of only 1 nm and rendering it unsuitable for industrial fields. Although the SGNN method possesses extremely high theoretical precision, its training depends entirely on simulated ground truth. This dependency makes it unable to adapt to label-free real physical environments, resulting in a lack of engineering practicality.

The PINN scheme proposed in this study achieves high-precision compensation of 0.23 nm without hardware modifications, striking a balance between cost and performance. Meanwhile, this scheme establishes a self-supervised paradigm that overcomes the limitations of simulation. It possesses field adaptability for blind identification on label-free real data, maintaining excellent robustness even under high noise of −12.29 dB.

## 5. Conclusions

This paper proposes a self-supervised intelligent calibration and error compensation method for heterodyne interferometric sensing systems, driven by a PINN incorporating frequency-domain priors. By integrating frequency-domain prior knowledge with physical hard constraints, the proposed method achieves the precise identification of nonlinear error parameters and the reliable reconstruction of error curves in a self-supervised manner. Experimental validations indicate that, even after removing low-frequency environmental drift and trend terms, the Pearson correlation coefficient between the network predictions and the ground truth remains above 0.98 (reaching up to 0.9939) under noise backgrounds with an effective SNR as low as −12.29 dB. This demonstrates the successful extraction of physical features from sensing signals heavily submerged in noise. After compensation, the RMS value of the nonlinear error is significantly reduced from 1.90 nm to 0.23 nm, and the compensation rate reaches up to 88.13%, realizing sub-nanometer measurement precision. Further analysis reveals a positive correlation trend between the compensation rate and the SNR, indicating that the post-compensation residual errors are primarily dominated by random noise, whereas the deterministic nonlinear harmonic components have been effectively eliminated by the physical model. These results not only validate the effectiveness of the proposed method under varying noise levels but also highlight the performance advantage of the PINN architecture in accurately extracting physical laws under strong noise backgrounds, providing a highly reliable solution for the error self-characterization and intelligent calibration of heterodyne interferometric industrial sensors.

## Figures and Tables

**Table 1:** Comparison of PINN predictions against ground truth and summary of nonlinear error compensation rates.

	Input SNR (dB)	Pearson Correlation Coefficient	Original Nonlinear Error RMS (nm)	Compensated Nonlinear Error RMS (nm)	Error Compensation Rate (%)
10 mm	−8.09	0.9939	1.9	0.23	88.13%
100 mm	−12.29	0.9817	1.9	0.37	80.32%

**Table 2:** Summary of comparison results between the nonlinear error outputs of the three models and the ground truth curves.

Model	RMSE (nm)	Pearson Correlation Coefficient
Model A	0.62	0.97
Model B	1.91	0.00
Model C	5.94	0.43

**Table 3:** Comparison of performance and characteristics between the proposed method and existing methods.

Method	Accuracy (nm)	Hardware Requirements	Ground Truth Label Dependency	Noise Robustness	In Situ Applicability
SGNN [32]	7.80 × 10^−3^	N/A	Yes	Low	No
Iterative DPLL [18]	0.80	Moderate	N/A	Moderate	Yes
DFNN [31]	≈1.00	High	Yes	Low	No
Proposed PINN	0.23	Low	No	High	Yes

## Data Availability

The original contributions presented in this study are included in the article. Further inquiries can be directed to the corresponding author.

## References

[1] Huang G., Cui C., Lei X., Li Q., Yan S., Li X., Wang G. (2025). A Review of Optical Interferometry for High-Precision Length Measurement. Micromachines.

[2] Yin Y., Sun X., Liu Y., Yin S., Zhao J., Mao Z., Wang S., Li Z. (2025). High-precision 12-times optical subdivision heterodyne laser interferometer for short-distance measurement. Opt. Express.

[3] Wang Y., Li J., Feng Q., Han S., Wang X., Fu W., Zhou M. (2025). Dynamic filling and prediction of environmental compensation parameters for positioning error interferometry of CNC machine tools based on a dual hybrid neural network. Opt. Express.

[4] Lin C., Zhou S., Shi L., Yang Y., Wu G. (2024). Two-dimensional angle measurement with sub-arcsecond precision and MHz update rate using heterodyne interferometry with optical frequency comb. Opt. Lett..

[5] Jia P., Zhang B., Feng Q., Zheng F. (2020). Simultaneous Measurement of 6DOF Motion Errors of Linear Guides of CNC Machine Tools Using Different Modes. Sensors.

[6] Cui C., Gao L., Zhao P., Yang M., Liu L., Ma Y., Huang G., Wang S., Luo L., Li X. (2025). Towards multi-dimensional atomic-level measurement: Integrated heterodyne grating interferometer with zero dead-zone. Light Adv. Manuf..

[7] Fu H., Wang Y., Hu P., Tan J. (2018). Nonlinear Errors Resulting from Ghost Reflection and Its Coupling with Optical Mixing in Heterodyne Laser Interferometers. Sensors.

[8] Quenelle R. (1983). Nonlinearity in interferometric measurements. Hewlett-Packard J..

[9] Bobroff N. (1987). Residual errors in laser interferometry from air turbulence and nonlinearity. Appl. Opt..

[10] Chen P., Hu P.C., Ding X.M., Tan J.-B. (2015). Current ways and means for reduction or elimination of periodic nonlinearity in heterodyne interferometer. Proc. SPIE Int. Soc. Opt. Eng..

[11] Fu H., Ji R., Hu P., Wang Y., Wu G., Tan J. (2018). Measurement Method for Nonlinearity in Heterodyne Laser Interferometers Based on Double-Channel Quadrature Demodulation. Sensors.

[12] Lu Z., Zhang Y., Liang Y., Tan J. (2018). Measuring the laser polarization state and PBS transmission coefficients in a heterodyne laser interferometer. IEEE Trans. Instrum. Meas..

[13] Guo J., Zhang Y., Shen S. (2000). Compensation of nonlinearity in a new optical heterodyne interferometer with doubled measurement resolution. Opt. Commun..

[14] Straube G., Calderón J.S.F., Ortlepp I., Füßl R., Manske E. (2021). A Heterodyne Interferometer with Separated Beam Paths for High-Precision Displacement and Angular Measurements. Nanomanuf. Metrol..

[15] Lawall J., Kessler E. (2000). Michelson interferometry with 10 pm accuracy. Rev. Sci. Instrum..

[16] Heydemann P.L.M. (1981). Determination and correction of quadrature fringe measurement errors in interferometers. Appl. Opt..

[17] Wu C.M., Su C.S. (1996). Nonlinearity in measurements of length by optical interferometry. Meas. Sci. Technol..

[18] Xie J., Yan L., Chen B., Zhang S. (2017). Iterative compensation of nonlinear error of heterodyne interferometer. Opt. Express.

[19] Bridges A., Yacoot A., Kissinger T., A Humphreys D., Tatam R.P. (2021). Correction of periodic displacement non-linearities by two-wavelength interferometry. Meas. Sci. Technol..

[20] Li Y., Dieussaert E. (2023). A compensation method for nonlinearity errors in optical interferometry. Sensors.

[21] Liu X., Peng W., Zhang X., Zhao X., Zhou W., Yao W., Chen X. (2025). Enhancing deep learning-based field reconstruction with a differentiable learning framework. Nat. Mach. Intell..

[22] Rehman A.U., Jiao W., Jiang Y., Wei J., Sohaib M., Sun J., Rehman K.U., Chi Y. (2025). Deep learning in industrial machinery: A critical review of bearing fault classification methods. Appl. Soft Comput..

[23] Chen C., Deng Y., Qian J., Ma H., Ma L., Wu J., Wu H. (2025). Deep learning-based inversion framework for fractured media characterization by assimilating hydraulic tomography and thermal tracer tomography data: Numerical and field study. Eng. Geol..

[24] Zheng S., He J., Liu C., Shi Y., Lu Z., Feng W., Ju F., Wang J., Zhu J., Min Y. (2024). Predicting equilibrium distributions for molecular systems with deep learning. Nat. Mach. Intell..

[25] Meng Z., Guan Z., Yu S., Wu Y., Zhao Y., Shen J., Lim C.C., Chen T., Yang D., Ran A.R. (2025). Non-invasive biopsy diagnosis of diabetic kidney disease via deep learning applied to retinal images: A population-based study. Lancet Digit. Health.

[26] Gao W., Yao W., Wang J., Mou H., Li Y., Zhao X., Duan J., Zou Y., Yao Y., Xu X. (2025). High-precision large-scale optical phased array calibration using physics-informed neural network with transfer learning. J. Light. Technol..

[27] Zhang L., Shi Z., Cheng M.M., Liu Y., Bian J.-W., Zhou J.T., Zheng G., Zeng Z. (2019). Nonlinear regression via deep negative correlation learning. IEEE Trans. Pattern Anal. Mach. Intell..

[28] Bolibar J., Rabatel A., Gouttevin I., Zekollari H., Galiez C. (2022). Nonlinear sensitivity of glacier mass balance to future climate change unveiled by deep learning. Nat. Commun..

[29] Li H., Ren T., Zhao C. (2025). A physics-informed neural network for non-linear laser absorption tomography. J. Quant. Spectrosc. Radiat. Transf..

[30] Gao S., Li Z., Brand U. (2024). Neural Network Approach for Modelling and Compensation of Local Surface-Tilting-Dependent Topography Measurement Errors in Coherence Scanning Interferometry. Metrology.

[31] Heo G.H., Lee W.R., You K.H. (2008). Adaptive Error Compensation of Heterodyne Laser Interferometer using DFNN. Trans. Korean Inst. Electr. Eng..

[32] Olyaee S., Ebrahimpour R., Hamedi S. (2010). Modeling and compensation of periodic nonlinearity in two-mode interferometer using neural networks. IETE J. Res..

[33] Hou W., Zhang Y., Le Y., Ju A. (2012). Elimination of the Nonlinearity of Heterodyne Displacement Interferometers. Chin. J. Lasers.

[34] Wu C.M., Deslattes R.D. (1998). Analytical modeling of the periodic nonlinearity in heterodyne interferometry. Appl. Opt..

